# A simulation of geographic distribution for the emergence of consequential SARS-CoV-2 variant lineages

**DOI:** 10.1038/s41598-022-14308-5

**Published:** 2022-06-15

**Authors:** Tetsuya Akaishi, Tadashi Ishii

**Affiliations:** 1 Department of Education and Support for Regional Medicine, Tohoku University Hospital, Seiryo-machi 1-1, Aoba-ku, Sendai, Miyagi 980-8574 Japan; 2 COVID-19 Screening Test Center, Tohoku University, Sendai, Japan

**Keywords:** Microbiology, Virology

## Abstract

The coronavirus disease 2019 (COVID-19) pandemic has been facilitated by the intermittent emergence of consequential variant strains. This study evaluated the geographic disproportionality in the detection of consequential variant lineages across countries. As of November 2021, a total of 40 potentially consequential SARS-CoV-2 variant lineages have been identified. One-hundred repeated simulations that randomly produced consequential variants from overall COVID-19 cases worldwide were performed to evaluate the presence of geographical disproportion in the occurrence of consequential variant outbreaks. Both the total number of reported COVID-19 cases and the number of reported genome sequences in each country showed weak positive correlations with the number of detected consequential lineages in each country. The simulations suggest the presence of geographical disproportion in the occurrence of consequential variant outbreaks. Based on the random occurrence of consequential variants among COVID-19 cases, identified consequential variants occurred more often than expected in the United Kingdom and Africa, whereas they occurred less in other European countries and the Middle East. Simulations of the occurrence of consequential variants by assuming a random occurrence among all COVID-19 cases suggested the presence of biogeographic disproportion. Further studies enrolling unevaluated crucial biogeographical factors are needed to determine the factors underlying the suggested disproportionality.

## Introduction

Coronavirus disease 2019 (COVID-19), caused by the severe acute respiratory syndrome coronavirus 2 (SARS-CoV-2), remains the world’s largest public health concern in 2022^1^. As of April, 2022, more than half a billion of people have been infected by the virus, resulting in six million deaths worldwide^2,3^. Infection control measures including the distribution of COVID-19 vaccines have been implemented across worldwide from the relatively early phase of the pandemic^4^, but the global pandemic is still ongoing with sporadic emergence of consequential variants along with changed transmissibility or severity that require monitoring^5,6^.

The SARS-CoV-2 virus is a single-stranded positive-sense RNA virus with a genome size of approximately 29,900 bases (Wuhan-Hu-1 strain, GenBank Accession ID: NC_045512)^7,8^. The genome of SARS-CoV-2 includes a gene coding the nsp14 enzyme that repairs replication errors, realizing complex transcriptional and translational tasks with boosted replication fidelity^9,10^. However, coronaviruses have the longest genome sequences among RNA viruses, and errors during genome replication are common and diversified^11,12^. Until now, numerous gene mutations with amino acid replacement, gene insertions, or gene deletions have been reported in SARS-CoV-2^13,14^. Most mutations in the SARS-CoV-2 genome are known to have no notable positive effect on their transmissibility or survival^15,16^. As a result, many of these gene mutations are eventually eliminated from the environment. However, some mutations are consequential with resultant selective advantages, which can survive in the population and spread to be predominant in some populations based on natural selection or founder effects^17,18^.

As of the end of November 2021, a total of 40 potentially consequential variant lineages (33 variants with identified countries where they probably originated and 7 variants with unidentified countries where they originated), classified by the Phylogenetic Assignment of Named Global Outbreak (PANGO) have been detected worldwide and designated to be strains worth watching by the World Health Organization (WHO)^19,20^. As the emergence of consequential variants with enhanced transmissibility could certainly trigger a new outbreak in the invaded regions or countries, clarifying the possible mechanisms and factors that may facilitate the emergence of consequential variants are essential for controlling the pandemic^21^. Among the suspected factors that could potentially influence the infection dynamics and occurrence of consequential variant strains, biogeographical factors that may facilitate the evolutionary potential of the virus must be considered^22,23^. Therefore, this study aimed to evaluate and determine the biogeographic disproportionality in the emergence and detections of the potentially consequential variant lineages that required monitoring by different countries and biogeographic regions.

## Methods

### Study objectives and design

**Figure 1:**
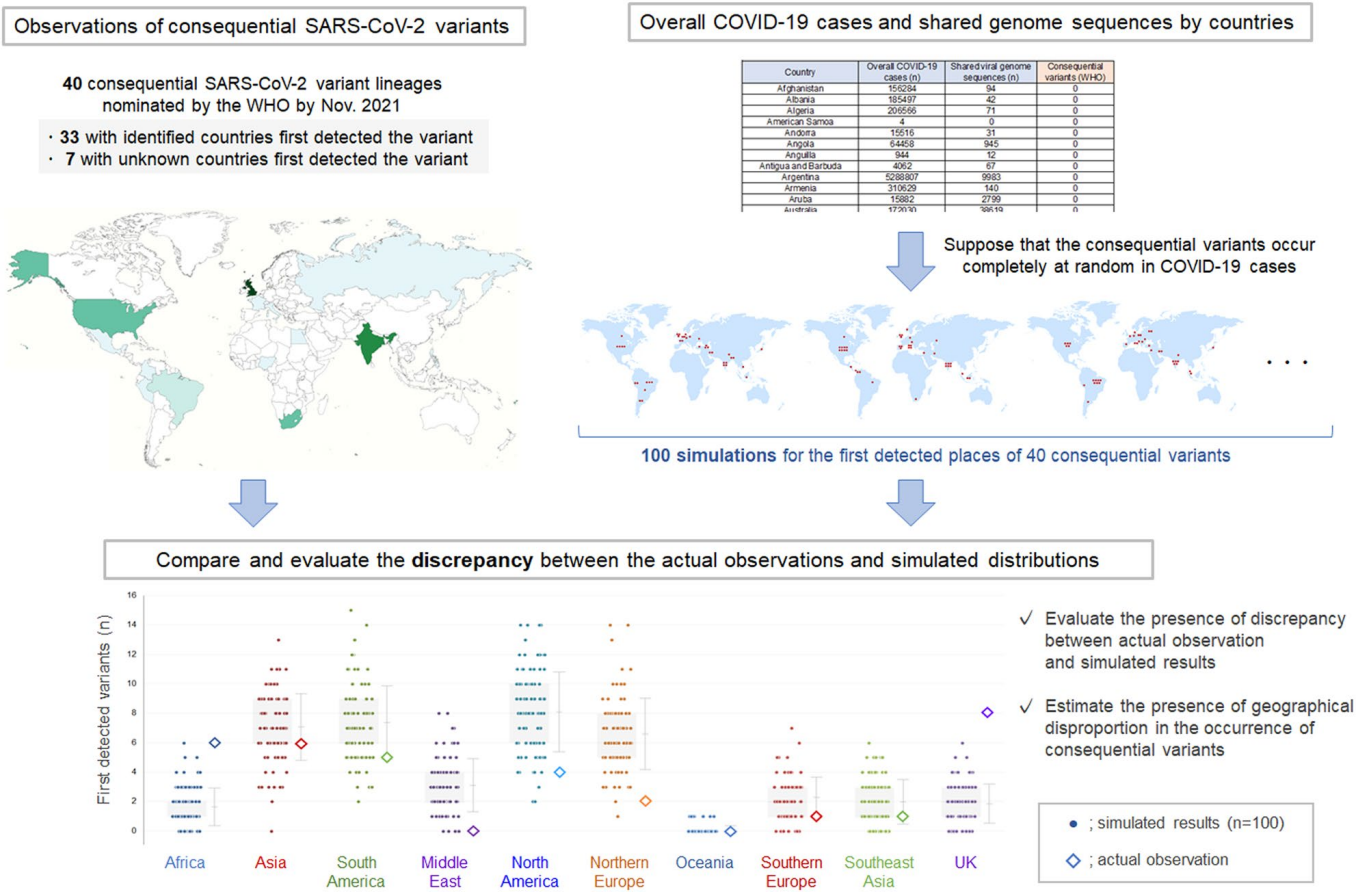
Flowchart depicting the research design of the present study. The main objective of the present study was to compare the actual observations of the first detected consequential SARS-CoV-2 variants and simulated numbers of the same variants in each country or biogeographical subregions worldwide. The simulations that randomly produced a total of 40 consequential variants from COVID-19 cases worldwide were repeated 100 times based on a null hypothesis that the occurrence of consequential variants would occur completely at random without geographical disproportions. Color maps were created using the MapChart software (https://www.mapchart.net). COVID-19, coronavirus disease 2019; SARS-CoV-2, severe acute respiratory syndrome coronavirus 2; WHO, World Health Organization.

The main objective of the present study was to compare the actual numbers and simulated numbers of the consequential SARS-CoV-2 variants first detected in each country or biogeographical regions worldwide. A flowchart depicting the objectives and research design of this study is presented in Fig. 1. Furthermore, the relationship between the overall number of COVID-19 cases or shared viral genome sequences in each country and the number of consequential SARS-CoV-2 variants first detected in each country was evaluated.

### Simulation of the occurrence of consequential lineages

A total of 100 simulations for the random occurrence of 40 consequential SARS-CoV-2 variants among overall COVID-19 cases in 223 areas and countries were performed, based on the assumption that the occurrence of consequential variant outbreaks would occur randomly among COVID-19 cases. More specifically, all countries were allocated a single or multiple unduplicated numbers between 1 and 10,000, the count of which correlates to the accumulated total number of COVID-19 cases by November 2021 in each country. The range of the allocated number (i.e., 1–10,000) was decided to avoid overestimating or underestimating the number of COVID-19 patients in smaller countries with fewer patients. The overall number of reported COVID-19 cases in each country was based on data reported by the Coronavirus Resource Center at Johns Hopkins University, USA (https://coronavirus.jhu.edu/). Then, an arbitrary number between 1 and 10,000 was randomly selected by using the random numbers produced based on the Mersenne Twister algorithm^24^. In each simulation run, a total of 40 random numbers between 1 and 10,000 were produced, and the corresponding country for each of the produced random value was listed. The simulations were repeated 100 times. The difference in the time-varying reproduction number between countries or regions, which is essential when considering the spatiotemporal spread of infection in each locality, was not considered in the present study because the simulations in the present study adopted a null hypothesis that consequential variants occur completely at random among the overall COVID-19 cases in the world without biogeographical disproportion^25,26^.

### Visual confirmation of the geographic disproportionality

The actual and simulated distributions for the occurrence of consequential SARS-CoV-2 variants were evaluated by plotting them on a world map to visually confirm the disproportionality of the distributions. The frequencies of occurrence between actual and simulated data were compared by country and biogeographic region. The biogeographic regions of the major prevailing countries were categorized in the following alphabetical order: (1) Africa, (2) Asia, (3) Central and South America, (4) Middle East, (5) North America, (6) Northern Europe (other than the United Kingdom [UK]) and Russia; (7) Oceania; (8) Southern Europe and the Mediterranean; (9) Southeast Asia; and (10) UK. The UK was separated from other European countries based on the country’s potentially distinct nature in terms of the number of reported COVID-19 cases and the first detected consequential SARS-CoV-2 variants from other European countries^27,28^. Armenia, Azerbaijan, and Georgia were categorized into Southern Europe. Turkey was categorized as the Middle East, and Egypt was categorized as African. Color maps of European and African countries scaled with the simulated or observed number of consequential variants first detected in each country were created using Map Chart Software (https://www.mapchart.net).

### Numbers of tested genome samples by countries

To check for possible bias derived from the different rates of tested genome samples among the infected patients in the countries worldwide, the number of tested genome samples before November 2021 in each country was further evaluated using data from the Global Initiative on Sharing Avian Influenza Data (GISAID; https://www.gisaid.org/). Correlations between the total number of COVID-19 patients, total number of shared genome samples, rate of sequenced cases among all patients, and number of potentially consequential variants first detected in each country were then evaluated.

### Statistical analyses

Correlations between the number of overall COVID-19 cases or submitted virus genome sequences and the first identified consequential SARS-CoV-2 variants in each country were evaluated using Spearman’s rank correlation coefficient (rho), based on the non-normal distributions of the variables. Tests for no correlation were performed to determine the statistical significance of the correlations. The median and interquartile range (IQR: 25–75 percentiles) of the expected number of consequential variants in each of the 10 regions were obtained from 100 simulations. Statistical significance was set at *p* < 0.05. Statistical analysis and the production of random numbers in the simulations were performed using R Statistical Software (version 4.0.5; R Foundation, Vienna, Austria).

## Results

### Detected potentially consequential SARS-CoV-2 variants

**Figure 2:**
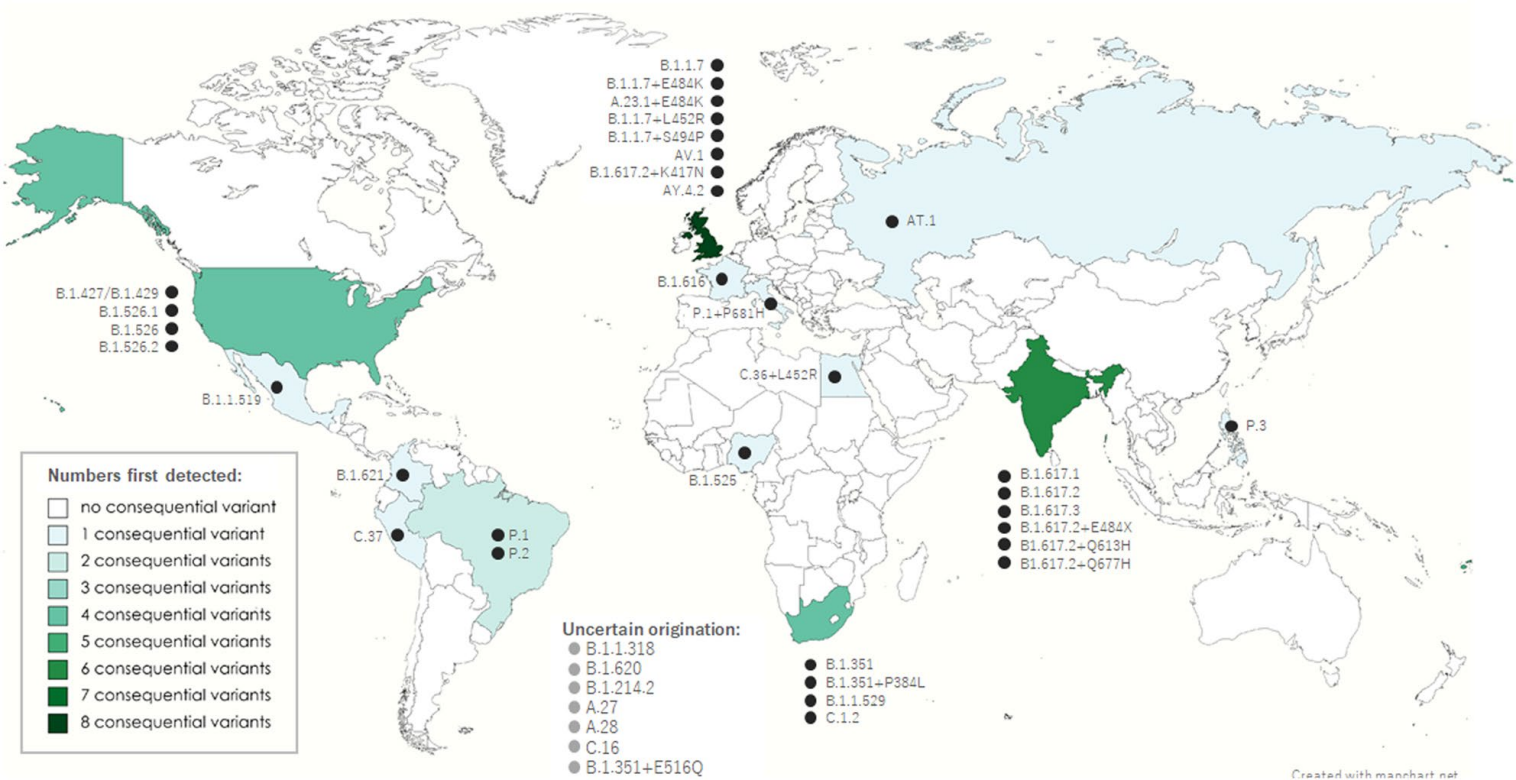
Potentially consequential SARS-CoV-2 variant lineages first detected in each country. The actual distribution of the potentially consequential SARS-CoV-2 variant lineages, first detected in each country by November 2021, is shown. Each black or gray dot represents each of the PANGO lineages that were nominated as variants that require careful attention by the WHO. The PANGO lineage annotation for each variant is described on the side of each dot. Color maps were created using the MapChart software.

By the end of November 2021, 40 variant strains with potentially changed transmissibility or severity had been detected worldwide. The WHO has nominated five of them (B.1.1.7 Alpha, B.1.351 Beta, P.1 Gamma, B.1.617.2 Delta, and B.1.1.529 Omicron) at least once as the Variants of Concern (VOC) and two (B.1.621 Mu and C. 37 Lambda) as Variants of Interest (VOI)^20,29^. Others are nominated as variants under monitoring (VUM) or de-escalated variants that dropped off from the prioritized watching list. The geographic distribution of the 33 variants with originating countries, which usually correspond to countries where the variants were first detected, is shown in Fig. 2. Notably, the country that first detected a variant that could differ from the origin country of the variant, such as the P.1 Gamma variant, which was first detected in travelers returning from Brazil to Japan^30,31^.

**Figure 3:**
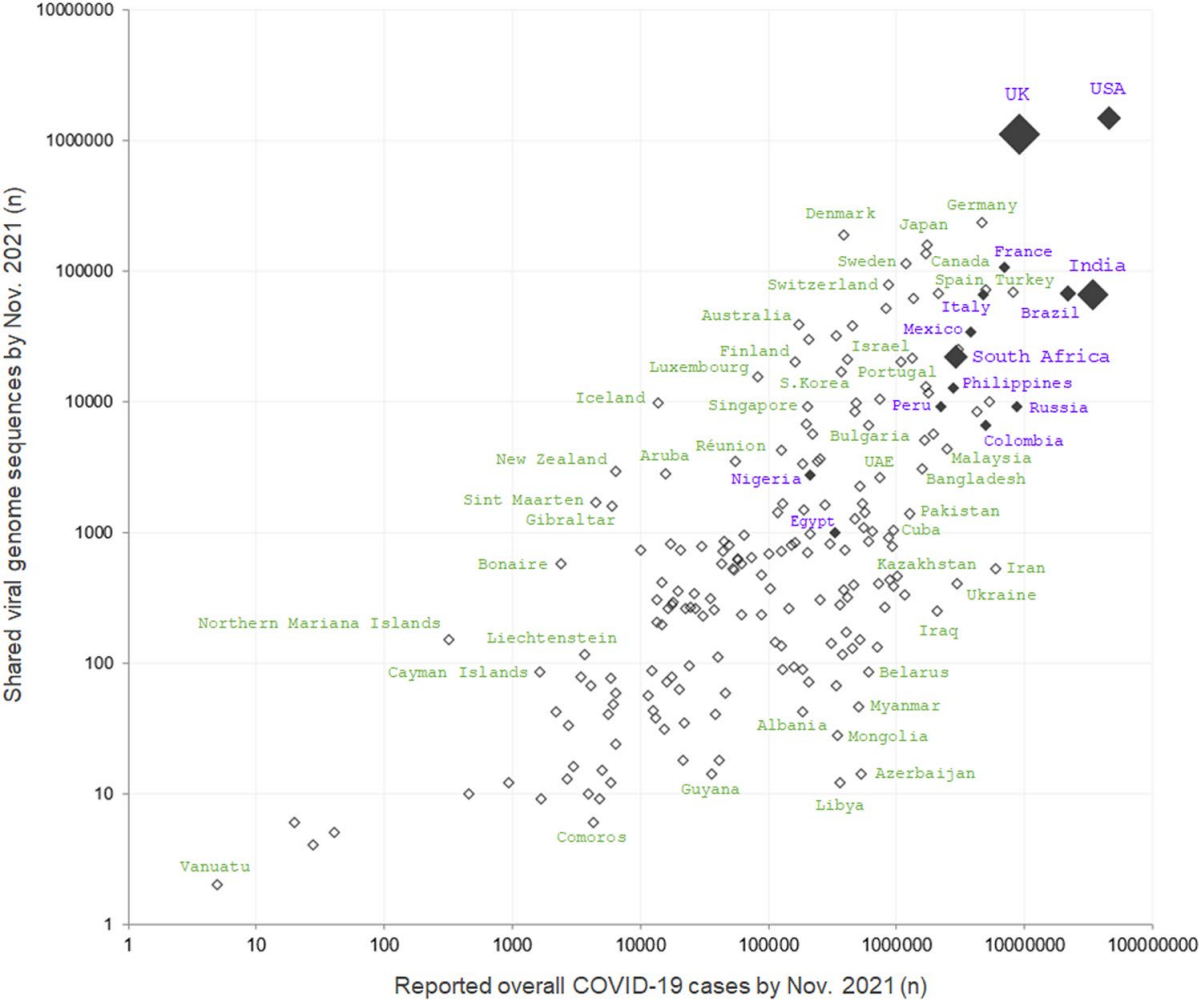
Scatterplot with the numbers of reported COVID-19 cases, shared genome sequences, and detected consequential variants in each country. A scatterplot of the number of reported COVID-19 cases and shared SARS-CoV-2 genome sequences in each country is shown. Each empty diamond represents a country with no consequential SARS-CoV-2 variant lineages first detected in the country. Each filled black diamond represents a country with at least one consequential lineage first detected, with its size proportional to the number of detected consequential lineages. The countries with purple font have at least one consequential SARS-CoV-2 variant first detected in them, whereas others with green font have no consequential variant first detected in them. The figure were created using Microsoft Office Excel 2016 software (https://www.microsoft.com).

Of the 223 areas and countries, the number of consequential variant lineages in each country was weakly correlated with the total number of COVID-19 cases (rho =  + 0.364; *p* < 0.0001) and number of shared genome sequences in each country (rho =  + 0.338; *p* < 0.0001). However, these correlations were weakened by a large number of countries without first detected consequential variants. To further investigate the impact of the number of overall COVID-19 cases and shared genome sequences on the number of confirmed consequential variant lineages in each country, a scatterplot was constructed using these variables in each country (Fig. 3). Each plot in the figure represents a single country, with the size proportional to the number of consequential lineages originating in each country. This distribution implied that the number of overall COVID-19 cases would contribute more to the detection of consequential variants than the number of shared viral genome sequences.

### Simulations for the random occurrence of consequential variants

**Figure 4:**
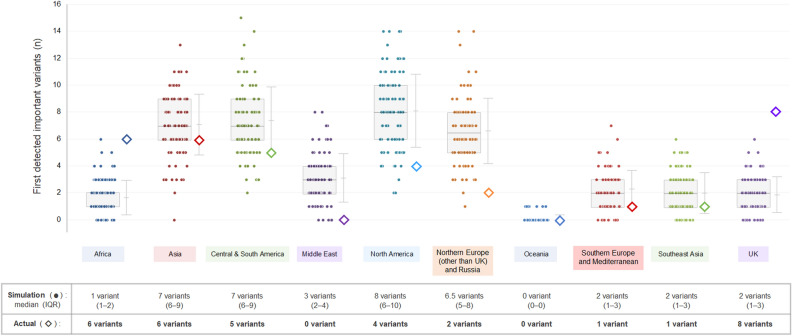
Simulations for the number of consequential variant outbreaks in each biogeographic region. The results of the 100 simulations for obtaining the expected number of consequential variant outbreaks supposing a random occurrence of consequential variants from overall COVID-19 cases are shown with filled dots in each of the 10 biogeographic regions. In addition to each grouped scatterplot, the actual number of consequential variants first detected in each region by November 2021 is shown with a large empty diamond. The median and interquartile range for the simulated numbers of consequential variants in each region are listed below the grouped scatterplots. The detection of consequential variants was higher than expected in the UK and Africa, and lower in the Middle East and European countries other than the UK. UK, United Kingdom, USA, United States of America.

**Figure 5:**
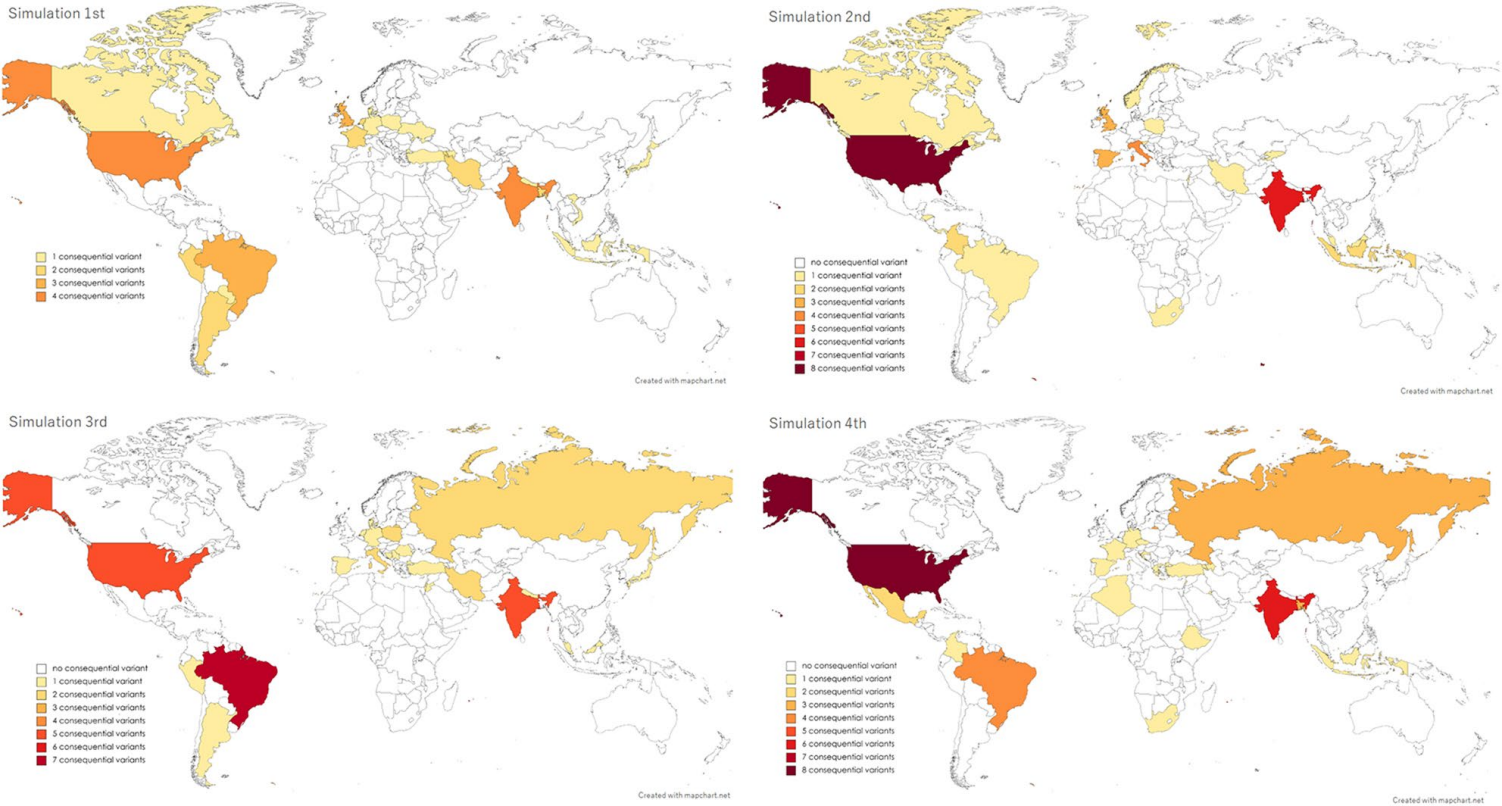
Examples of the obtained simulation results for the occurrence of consequential variant outbreaks. The results of the first four of the 100 simulations regarding the geographical distribution of the consequential variant outbreaks are shown. In the simulations, a random occurrence of a consequential variant lineage among all COVID-19 cases was assumed. Each filled dot represents the simulated occurrence of a consequential SARS-CoV-2 variant lineage. By comparing the obtained results with the actual distributions, as shown in Fig. 1, the actual numbers of the detected lineages in the areas of the Middle East and European countries other than the UK were lower than expected from the simulations, whereas the numbers in the UK and South Africa were higher than expected by the simulations. Color maps were created using the MapChart software.

**Figure 6:**
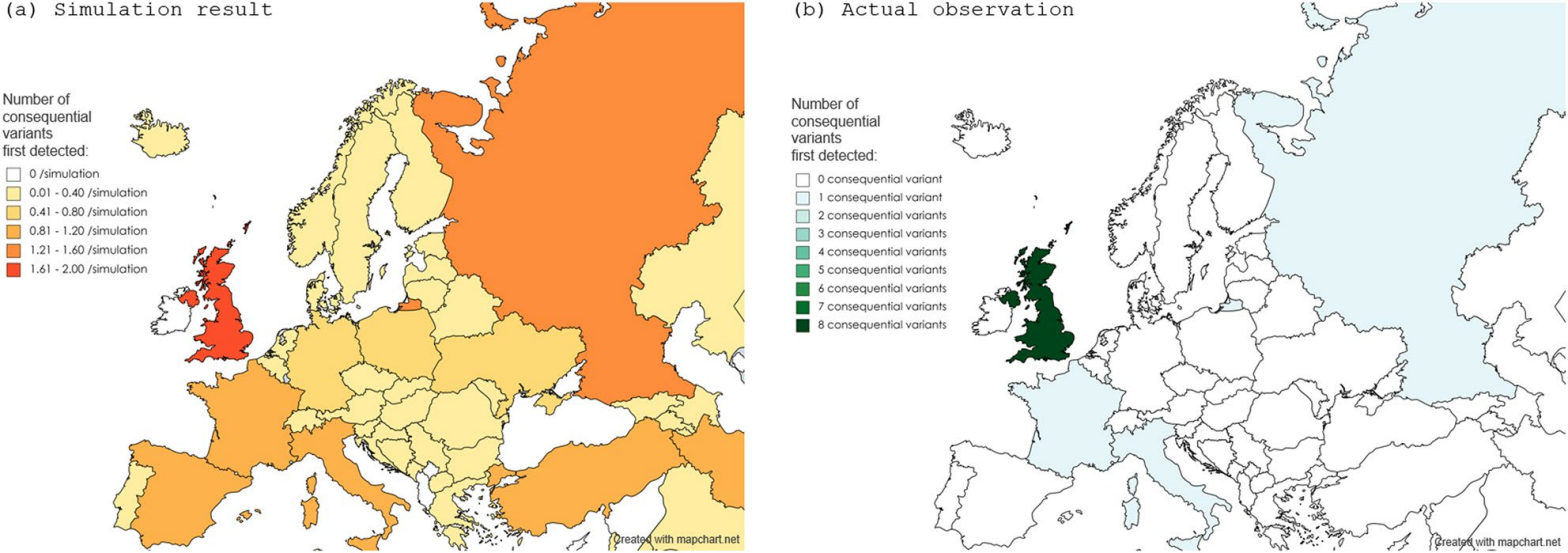
Map for simulated or observed number of consequential SARS-CoV-2 variant first detected in European countries. The simulated (**a**) or observed (**b**) number of consequential SARS-CoV-2 variants first detected in each European country is shown. The actual distribution with apparent geographical disproportion implies the presence of a threshold in the prevalence of the infection or an unknown biogeographical factor for the emergence and spread of the consequential variants. Color maps were created using the MapChart software.

**Figure 7:**
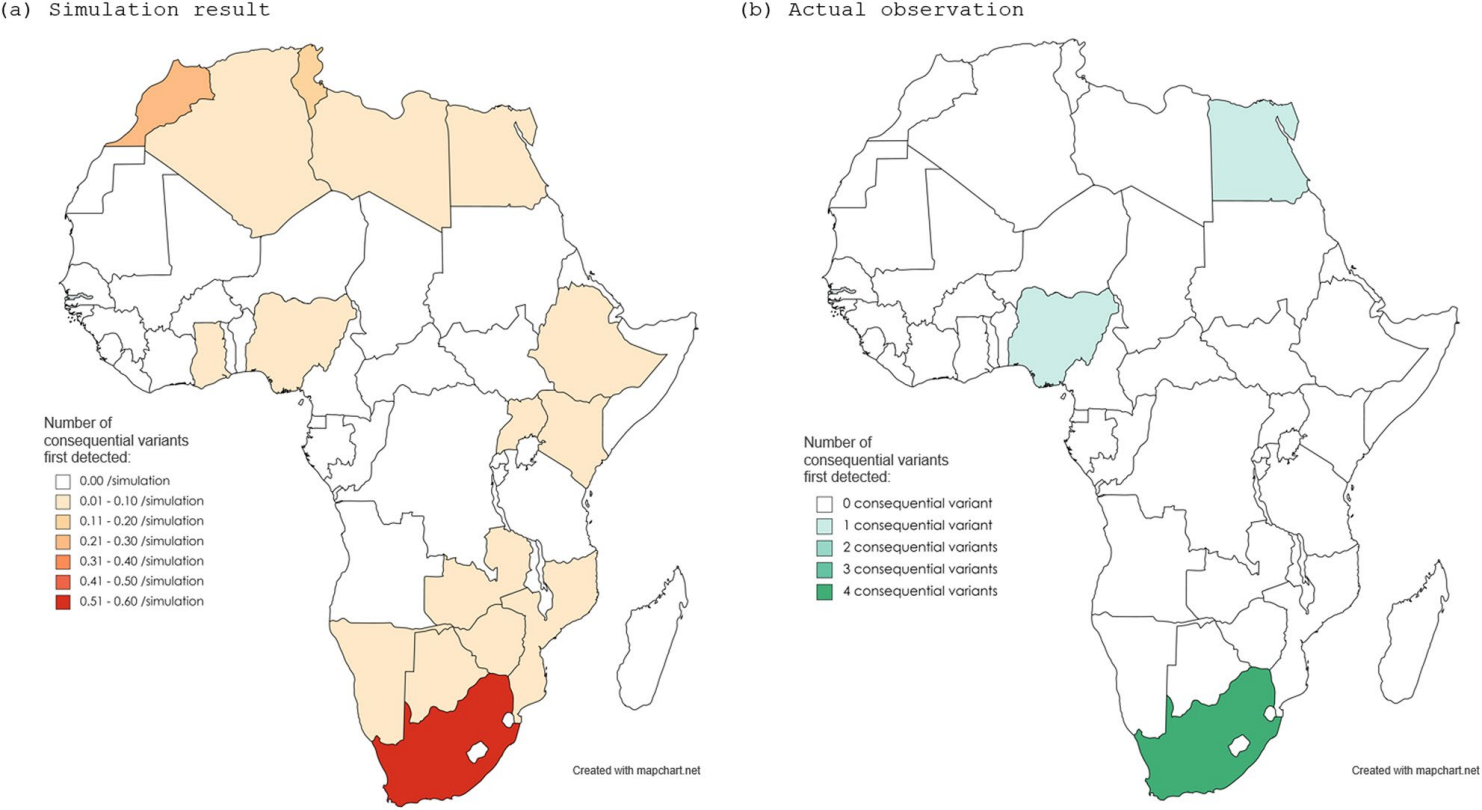
Map for simulated or observed number of consequential SARS-CoV-2 variant first detected in African countries. Similar to color maps in Europe, the presence of biogeographical disproportion for the emergence and spread of consequential variants has been suggested in African countries. Color maps were created using the MapChart software.

Based on the assumption that the emergence of variant lineages, irrespective of consequentiality, occurs randomly from all infected populations worldwide, 100 simulations for the occurrence of consequential variants were repeatedly performed. The expected number of consequential variants in each of the 10 biogeographic regions, together with the actual detected numbers in each region, is shown in Fig. 4. The number of detected consequential variants in the UK and Africa were suggested to be higher than those expected from the simulation data. To visually confirm the suggested geographical disproportion in the occurrence of consequential variant outbreaks, simulation data regarding the geographical distribution of consequential variant outbreaks with the first four simulations are shown in Fig. 5. These simulation data suggest that the emergence of consequential variants may not be random. The simulated distributions, compared with the actual distributions of consequential variants, implied a possible presence of geographical disproportion in the occurrence of consequential variants not only between the 10 biogeographical regions but also between the countries in each of the regions. For example, the geographical distribution of the simulated (a) or observed (b) consequential SARS-CoV-2 variants in each European country is shown in Fig. 6. Of the 4000 consequential variants produced in the 100 simulations, 1069 were from European countries. Among them, only 184 (17.2%) were from the UK. The difference between the simulated data and actual observed distribution may imply that a threshold in the prevalence of the infection or some biogeographical disproportion may exist for the emergence and spread of consequential SARS-CoV-2 variants in each country. Similar results were obtained for African countries (Fig. 7). Of the 4000 consequential variants produced in the 100 simulations, 164 were from African countries. Among these, 60 (36.6%) were from South Africa.

## Discussion

In the present study, the actual geographic distributions of the worldwide occurrence of potentially consequential SARS-CoV-2 variants were compared with 100-times simulated distributions, assuming a random occurrence of a consequential variant among overall COVID-19 cases. The strength of the simulation in the present study was that it enrolled all countries worldwide, even with small numbers of COVID-19 cases, not to underrepresent the contributions of countries with fewer COVID-19 cases. The simulation was repeated up to 100 times to obtain reliable data for the expected number of consequential variants first detected in each geographical region or country. The results suggest the presence of a discrepancy between the actual and simulated distributions. Such a geographical disproportion was implied in Europe, the Middle East, and Africa. In the Middle East and in European countries other than the UK, the actual numbers of first detected consequential variants were suggested to be higher than expected based on the random occurrence of consequential variants. Meanwhile, the actual numbers in the UK and Africa were suggested to be higher than expected, based on the random occurrence of consequential variants.

A possible explanation for the observed geographic disproportionality may be the difference in the performed frequencies of genome-wide analysis for SARS-CoV-2 genes between countries and regions. However, as implied by the data obtained from the Nextstrain Study Group, this possibility seems less likely. The results of the present study imply that the number of overall COVID-19 cases would contribute more to the number of first detected consequential variants than the number of shared viral genome sequences in each country. In regions where geographical disproportions were suspected, most of the major constituent countries have performed and reported data with adequate qualities regarding the whole genome sequences from the early phase of the pandemic to evaluate the genetic diversity of SARS-CoV-2 in the regions^32–38^. Another possibility is that unevaluated factors that may produce biogeographical disproportion may have affected the occurrence and spread of the potentially consequential variant lineages. Conceivable factors may include host-side biological and genetic backgrounds (e.g., immunocompromised host), lifestyles in the locality, animals as possible natural reservoir hosts of the virus, and other unknown environmental and ecological factors^39–43^. These possibilities seem to be reasonable, as the virus replication and spread depend on host translation machinery^44,45^. Further studies are needed to determine whether such host-side factors with geographical disproportion behind the occurrence of consequential variants really exist.

This study had some limitations. First, the correctness of assuming that the number of occurrences of the variant lineages is proportional to the number of overall COVID-19 cases in the region is uncertain. The geographic distribution of consequential variants could be attributed to multiple factors that were not evaluated in this study, as discussed above. Moreover, the performance levels of diagnostic screening tests or contact tracing may differ significantly between countries, making the reported numbers of overall COVID-19 cases across countries worldwide may be underestimated in many countries. Another limitation is that the exact relationship between the frequency of viral genome sequencing and the number of consequential variants first detected in each country is uncertain. These uncontrolled factors should be adequately considered and adjusted in future studies.

## Conclusions

The results of the simulations in the present study demonstrated that there may be geographical disproportion in the occurrence of consequential SARS-CoV-2 variants between biogeographical regions and countries. This finding may imply that some unknown host-side factors may exist behind the emergence and spread of the new potentially consequential SARS-CoV-2 variants, and that the consequential variant outbreak may not occur completely at random among COVID-19 patients.

## Supplementary Information

Supplementary Information.

## Data Availability

All data generated or analyzed during this study are included in this published article and its supplementary information files (Supplementary Table 1).
